# Sustainable cannabinoids purification through twin-column recycling chromatography and green solvents

**DOI:** 10.1007/s00216-024-05332-7

**Published:** 2024-05-15

**Authors:** Greta Compagnin, Chiara De Luca, Chiara Nosengo, Martina Catani, Alberto Cavazzini, Giorgia Greco, Yannick Krauke, Simona Felletti

**Affiliations:** 1 Department of Chemical, Pharmaceutical and Agricultural Sciences, University of Ferrara, via L. Borsari 46, Ferrara, 44121 Italy; 2 KNAUER Wissenschaftliche Geräte GmbH, Hegauer Weg 38, Berlin, 14163 Germany; 3 Council for Agricultural Research and Economics, CREA, via della Navicella 2/4, Rome, 00184 Italy; 4 Department of Environmental and Prevention Sciences, University of Ferrara, via L. Borsari 46, Ferrara, 44121 Italy

**Keywords:** Recycling chromatography, Cannabinoids, *Cannabis sativa* L., THC depletion, Green solvent

## Abstract

**Abstract:**

In the present study, twin-column recycling chromatography has been employed for the purification of a *Cannabis* extract by using a green solvent, ethanol, as the mobile phase. In particular, the complete removal of the psychoactive tetrahydrocannabinol (THC) from a *Cannabis* extract rich in cannabidiol (CBD) was achieved under continuous conditions. The performance of the method, in terms of compound purity, recovery, productivity and solvent consumption, was compared to that of traditional batch operations showing the potential of the twin-column recycling approach. The employment of a theoretical model to predict the band profiles of the two compounds during the recycling process has facilitated method development, thus further contributing to process sustainability by avoiding trial and error attempts or at least decreasing the number of steps significantly.

**Graphical abstract:**

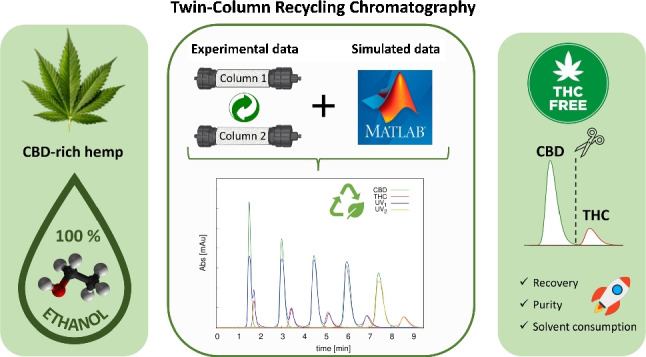

**Supplementary Information:**

The online version contains supplementary material available at 10.1007/s00216-024-05332-7.

## Introduction

*Cannabis sativa* L. has become one of the main research topics in the medicinal, pharmaceutical and nutraceutical field, thanks to its various beneficial properties and therapeutic effects. The most known cannabinoids found in *Cannabis* are cannabidiol (CBD) and tetrahydrocannabinol (THC), but their relative abundance depends on the *Cannabis* chemotype. As an example, chemotype I has a higher THC content (THC/CBD>1) while in chemotype III CBD is dominant, with a small content of THC (<0.2%) [1, 2].

The psychotropic effect of THC and its abuse have generated, in many countries, legal limitations for the business of *Cannabis* products [3–5], which however is fastly growing. Based on these restraints, different depletion processes have been implemented to reduce or eliminate THC from *Cannabis* extracts. These techniques are based on multiple steps, often combining low pressure and low efficiency flash chromatography, high performance liquid chromatography (HPLC) which can work on normal (NP) or reversed (RP) phase, or different dissolution and crystallisation steps [6–13].

Normal phase elution mode has been successfully used for cannabinoids purification, with optimal results and fast separations [8, 14]. In addition, cannabinoid solubility in solvents such as hexane or heptane is significantly large (e.g. CBD solubility in hexane is >100 mg/mL), allowing the loading onto the column of high amounts of feed. This is important when it comes to the purification of these compounds since the larger the solubility, the larger the amount of material that can be loaded into the column and processed.

In the last few years, increasing attention has been paid on the replacement of harmful organic solvents (e.g. chloroform, dichloromethane, methanol, hexane, pentane, heptane or acetonitrile [15–17]) with greener and more ecofriendly alternatives. Moreover, the adoption of efficient and cutting-edge techniques with low energy requirements and low solvent consumption to account for the principles of green chemistry and to improve process sustainability has been also considered [18]. Among green solvents, ethanol (EtOH) seems to be particularly promising. Ethanol, indeed, has been classified as a fully “recommended” solvent, with no negative effects on both environment and humans [19, 20]. Furthermore, its low boiling point favours solvent evaporation and removal with small energy requirements if compared to water.

The use of pure EtOH as mobile phase in RPLC for the purification of cannabinoids is advantageous from several viewpoints. First, EtOH is commonly used as extraction solvent for cannabinoids. This permits the direct injection of the sample after extraction onto the chromatographic column without further evaporation and solubilisation steps. Secondly, purified samples can then be used to prepare commercial big selling products based on cannabis, such as personal care products or even food or beverages. EtOH, indeed, is labelled as a Generally Recognised as Safe (GRAS) substance by the Food and Drug Administration (FDA) to be used in food products. On the other hand, it has been shown that ethanolic mobile phases in RPLC are characterised by very high elution strength which provokes reduced analyte retention and often poor resolution between analytes. When employed as mobile phase for the separation of *Cannabis* extracts, for instance, EtOH leads to two groups of not fully resolved cannabinoids: the first group contains early eluting compounds (e.g. CBD and cannabigerol, CBG), while the second group contains more strongly retained compounds (e.g. THC and cannabichromene, CBC). To alleviate this drawback and obtain acceptable resolution between these two groups of cannabinoids, different strategies can be adopted. To decrease the flow rate is not an optimal method, since its success clearly depends on the initial selectivity and peak broadening, hence it cannot be applied to all samples. Indeed, when changing the flow rate, selectivity remains constant and only an increase in column efficiency will lead to a change in the resolution between two peaks. Following van Deemter curves, optimal efficiency is usually obtained at low flow rates, which translates in longer run times. An easy approach to consider is to use longer columns. However, the length of HPLC columns is limited to a maximum of usually 30 cm, mainly due to instrumental impediments related to backpressures generated by longer columns especially in RP conditions. High temperatures could be the key to reduce column backpressure by decreasing solvent viscosity. Nevertheless, this hypothesis is rarely applicable since heat is responsible for the degradation of cannabinoids. An alternative strategy to overcome the co-elution issues could be the recycling of either the entire peak or portions of the peak. The principle of peak recycling process is to re-inject the eluted target peak into the same column (closed loop recycling), or into a second column (alternative pumping recycling), forming a circuit which can be repeated several times simulating one big column whose length is proportional to the number of switches performed and therefore increasing the resolution power of the system [21].

The twin-column recycling operation was used in the past to separate polymer mixtures, through gel permeation chromatography, and to collect optically active compound with chiral liquid chromatography [22, 23]. Even though the interest for this mode of operation is lower compared to, e.g., continuous or semicontinuous processes, in some cases it represents a very workable and practical solution, especially thanks to the ease of the experimental setup implementation and efficiency to handle co-elutions [24].

In this work, for the first time, twin-column recycling chromatography was applied to a CBD-rich *Cannabis* extract for the separation and the final depletion of THC using a totally green method, based on pure EtOH. Method performance in terms of productivity, solvent consumption, final purity and recovery have been compared with traditional single column batch separation. Method development was supported by theoretical modelling to determine the number of cycles to be repeated to reach the desired separation.

Results look very promising and suggest that the combination of theoretical predictions and multi-column chromatography with green mobile phase, can be employed for the purification of *Cannabis* as an efficient alternative to traditional techniques.

## Theory

### Principles of recycling chromatography

Recycling chromatography is a well-established technique, used in the past to separate polymers and chiral compounds [22, 23]. The principle is to increase the resolution between not fully resolved binary mixtures, avoiding the final re-mixing of the components. This is achieved by virtually increasing the column length to produce larger retention and separation performance through the subsequent re-injections of the peak(s) into the same column (closed loop recycling) or into a second identical column (alternative pumping recycling) [25]. Closed loop recycling (CLR) is considered a simple method, since it makes use of only one column, and the eluted target peak is re-injected into the same column through a pump by the switching of a 2-position valve. Its main disadvantage is the pump large dead volume, which largely contributes to the peak broadening, possibly causing the re-mixing of analyte peaks.

**Fig. 1:**
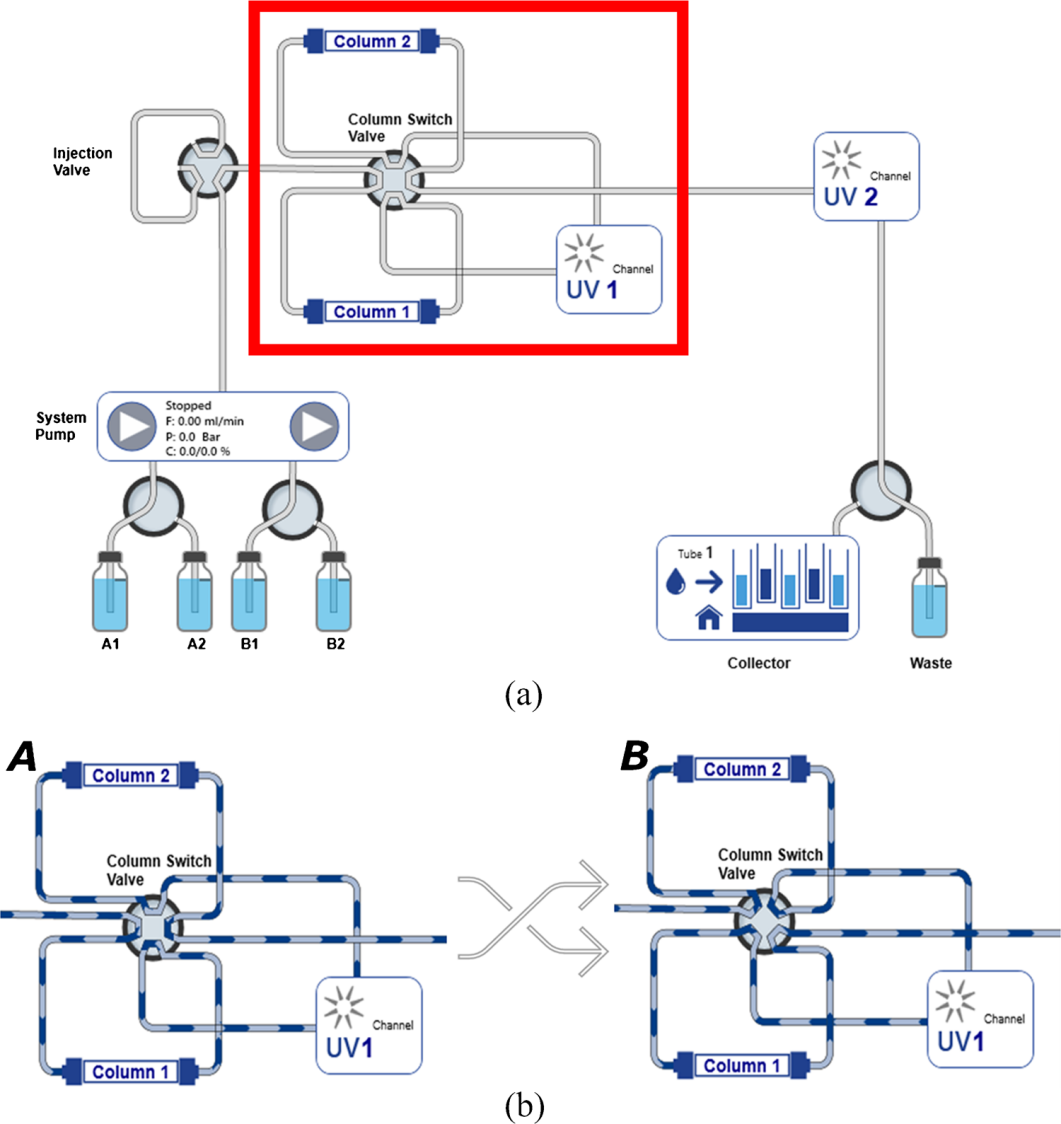
(a) Visualisation of alternative pumping recycling system by PurityChrom®6 software. In the red rectangle the recycling circuit is highlighted, which is made of two twin columns (column 1 and column 2) and a UV detector (UV_1_). (b) Representation of two flow paths generated by switching a 8-port-2-position valve. In configuration A the flow goes through column 1 to UV_1_ to column 2. In configuration B the flow goes through column 2 to UV_1_ to column 1

Alternative pumping recycling (APR) is an improved design of CLR. In this setup, the target peak does not have to pass through the pump but is directly re-injected into a second twin column [26]. The outlet of one column is directly connected to the inlet of the second column through a 2-position valve (Fig. 1), so that the flow never stops. More in detail, in APR chromatography, the twin columns are connected by an 8-port-2-positions valve with an UV detector placed between them (Fig. 1a). Figure 1b shows the two flow paths that can be generated into the recycling circuit by switching the valve. Let’s assume that column 1 is the first column to be loaded with feed. At the beginning of the process, during feed loading and right after it, the columns are in position A according to Fig. 1b, where the mobile phase coming from the pump flows into column 1, to UV_1_ and then to column 2. So the peaks eluting from column 1 are immediately recycled into column 2, thereafter, the valve switches position and the system is now in position B, where the mobile phase goes from the pump into column 2, to UV_1_ and then to column 1. When the position of the valve changes from A to B or vice versa, one switch has been performed. At the end of the process, the two columns are simply connected in series and the eluate flows within both of them and both the detectors. Detector 2 is only used at this moment of the process, since during the cycles only detector 1 is used and is placed between the two columns.

The number of switches, $n_s$ is directly related to the number of columns *n* through $n=n_s+1$: when the sample is injected into the first column ($n=1$), no switches have been performed ($n_s=0$), after the first switch ($n_s=1$), the sample is then injected into the second column, thus experiencing $n=2$ columns, and so on. At the end of the cycle, the sample eluting from a column is injected into the other column without further switches, hence the total number of columns is $n_{tot}=n_s+2$.

The presence of the detector (UV_1_) makes it possible to monitor the separation in real time. Without the detector UV_1_, internal to the circuit, peaks are only detected at the end of the cycles (i.e. when the peaks leave both columns) by UV_2_. In this case, multiple individual runs with 0, 1, 2,..., $n_s$ switches must be performed to know the progress of the separation [27].

The parameters useful to quantify separation and purification performance are: resolution (*R*), purity ($P(\%)$), recovery ($Rec(\%)$), productivity and solvent consumption.

The resolution, after a passage through one column, is defined by retention volumes of the two species, $V_{1}$ and $V_{2}$, with $V_{1}$ the less retained and $V_2$ the most retained peaks, and the corresponding peak volume variances, $\sigma ^2_{1}$ and $\sigma ^2_{2}$ [25, 28]:

1 $$\begin{aligned} R=\frac{V_2-V_1}{2\big (\sigma _{1} + \sigma _{2}\big )} \end{aligned}$$

After the passage through *n* columns, retention volumes as well as peak variances are proportionally increased ($V_{i,n}=nV_i$ and $\sigma ^2_{i,n}=n\sigma ^2_{i}$), following:

2 $$\begin{aligned} R_n=\frac{n (V_2-V_1)}{2\big (\sqrt{n \sigma ^2_{1}}+\sqrt{n \sigma ^2_{2}}\big )}=\sqrt{n}R \end{aligned}$$

The purity of each analyte is defined as the ratio of the peak target area ($A_i$) and the total area of the chromatogram ($A_{total}$):

3 $$\begin{aligned} P\,(\%) = \frac{A_i}{A_{total}}\cdot 100 \end{aligned}$$

The recovery is defined as the mass of target analyte ($m_i$) collected with respect to the total injected target mass ($m_{i,total}$):

4 $$\begin{aligned} Rec\,(\%) = \frac{m_i}{m_{i,total}}\cdot 100 \end{aligned}$$

The productivity represents the ratio of amount of target analyte ($m_i$) collected per unit of run time (*time*):

5 $$\begin{aligned} Productivity\, \text {(mg/h)} = \frac{m_i}{time} \end{aligned}$$

In addition to these parameters, solvent consumption is another metric which describes the quality of the process in terms of greenness. It is determined as ratio of the amount of solvent used ($V_{tot}$) during the whole run and the mass of target product collected ($m_{i}$).

6 $$\begin{aligned} Solvent\, Consumption\, \text {(mL/mg)} = \frac{V_{tot}}{m_{i}} \end{aligned}$$

It is worth pointing out that productivity and solvent consumption take into account the total separation time (i.e. the time required to the analytes to pass through *n* columns) and the total volume of solvent needed for *n* column passages.

### Theoretical band prediction

A general problem associated to recycling chromatography is that the number of switches is limited to a maximum. Switches can be made only until the spatial width of the separation zone reaches the column length [29, 30], otherwise the so-called re-mixing of the two peaks occurs. The switching of the valve to another position will then cut off part of the peak that exceeds the separation space of one column [25, 31]. Within this framework, an accurate prediction of the behaviour of band profiles depending on the number of switches (or column passages) would be advantageous. This can be achieved through a simple MATLAB code which provides numerical solutions of the Equilibrium-Dispersive (ED) Model of chromatography [32, 34]. Within this model, the accumulation of material in a thin slice of column is described through a differential mass balance equation (MBE):

7 $$\begin{aligned} \frac{\partial C}{\partial t}+F\frac{\partial q}{\partial t}+u\frac{\partial C}{\partial z}=D_a\frac{\partial ^2 C}{\partial z^2} \end{aligned}$$

with *C* and *q* the concentrations of the analyte in the mobile and stationary phases, $F=(1-\epsilon _t)/\epsilon _t$ the phase ratio and $\epsilon _t= V_0/V_{col}$ the total porosity of the column (with $V_0$ and $V_{col}$ the thermodynamic void volume and the column volume, respectively). Mobile and stationary phases are assumed to be constantly in equilibrium and an apparent dispersion coefficient, $D_a$, encloses all the contributions to band broadening:

8 $$\begin{aligned} D_a=\frac{uL}{2N} \end{aligned}$$

where *u* is the mobile phase linear velocity, *L* the length of the column and *N* the number of theoretical plates.

In order to numerically solve Eq. 7, a finite difference method and an isotherm model, expressing *q* as a function of *C*, must be chosen. In this work, a linear isotherm is considered, where $q=aC$. *a* is the Henry’s constant and it is linked to the retention factor, *k*, through $a = k/F$. Finite difference methods state that the continuous plane (*z*, *t*) of space and time is given by a grid made of a defined number of equal segments of width *h* for space and $\tau$ for time and that a finite difference term replaces each term of Eq. 7 (for further details the reader is referred to [32]). The application of the forward-backward scheme leads to:

9 $$\begin{aligned} C_{n+1}^j=C_n^j+\frac{h}{\tau }\big (G^j_n-G_n^{j-1}\big ) \end{aligned}$$

with $G(C)=(C+Fq)/u$. With this method it is possible to predict band profiles at successive time intervals ($j-1$ and *j*) and to calculate analyte concentration at $n+1$ space position ($C_{n+1}$), knowing the concentration at *n* position ($C_n$).

To perform the precise simulation of peak profiles, column and sample characteristics are needed. Geometrical characteristics of the column, such as dimensions and porosity, are easily obtained. The sample elution behaviour can be obtained through peak fitting of an experimental chromatogram with the exponentially modified Gaussian (EMG) function. EMG function is able to model real chromatographic peaks through the convolution of a Gaussian function with an exponential decay function. The advantage of this empirical model is its double use to represent both symmetrical and tailed peaks [35]. It is defined as follows for the *i*th component:

10 $$\begin{aligned} C_i(t)= \frac{1}{2\tau _i}exp\Bigg [\frac{\sigma ^2_i}{2\tau ^2_i}-\frac{t-t_{R,i}}{\tau _i}\Bigg ]erfc\Bigg [\frac{1}{\sqrt{2}}\Bigg (\frac{t-t_{R,i}}{\sigma _i}+\frac{\sigma _i}{\tau _i}\Bigg )\Bigg ] \end{aligned}$$

where $t_R$ is the retention time, $\sigma$ is the standard deviation, $\tau$ is the time constant of exponential decay function and *erfc* is the complementary error function.

Then, first ($\mu _1$) and second central ($\mu _2$) moments can be calculated [33]:

11 $$\begin{aligned}&\mu _1=\frac{\int _0^{100}C(t)t dt}{\int _0^{100}C(t)dt}\end{aligned}$$

12 $$\begin{aligned}&\mu _2=\frac{\int _0^{100}C(t)\big (t-\mu _1\big )^2 dt}{\int _0^{100}C(t)dt} \end{aligned}$$

The MATLAB code scheme is reported in SI (Fig. S1). Briefly, the simulation process consists of different steps: i) experimental injection of the sample; ii) peak fitting using EMG function; iii) determination of parameters ($t_R$, $\sigma$, $\tau$, plate number); iv) calculation of band profiles using EMG function and solving ED model; v) prediction of recycling chromatography by varying *n*.

The use of simulation programs permits to investigate in advance the maximum number of allowed switches to prevent peak overlapping between the two columns and the number of switches needed to achieve the best separation/resolution. This is a very important aspect to consider, especially from practical and industrial viewpoints, since it permits to avoid the trial and error strategy and to save time and resources, contributing to the green transition of chromatographic processes.

## Experimental

### Chemicals and solvents

CBD and $\Delta$ ^9^-THC standard solutions were from Cerilliant (Round Rock, Texas, USA). HPLC-grade solvents, including ethanol (EtOH), acetonitrile (ACN) and orthophosphoric acid (85%) were from SigmaAldrich (St. Louis, MO, USA).

The plant material from a non-psychoactive *Cannabis sativa* variety (chemotype III) was firstly decarboxylated at 140 ^∘^C for 1 h and then extracted by means of dynamic maceration at room temperature using ethanol as extraction solvent for 15 min. Sample was filtered with 0.2 $\mu$m PTFE filters prior to injection.

### Offline analysis

Offline measurements, used to evaluate purity and recovery of analytes, were performed under RP conditions using a 150 $\times$ 4.6 mm Eurospher II C18P column packed with 3 $\mu$m fully porous particles on an AZURA HPLC system (KNAUER, Berlin, Germany) equipped with a binary pump (10 mL head pump), a column thermostat, an autosampler and a photodiode array detector (DAD). Data acquisition, data handling and instrument control were performed by ClarityChrom CDS software. Mobile phases were a phosphate buffer solution at pH=2.2 and pure acetonitrile (ACN). The gradient program was set as follows: 0–7 min 75% ACN, 7–17 min from 75% to 90% ACN, 17–19 min 90% ACN, 19–22 min 75% ACN [11]. The wavelength was set at 228 nm. Injection volume was 5 $\mu$L. Calibration was performed using cannabinoid standards with known concentrations, ranging from 1 to 75 $\mu$g/mL.

### Recycling chromatography method

APR process was performed under RP conditions using two identical 150 $\times$ 8 mm Eurospher II C18 columns packed with 10 $\mu$m fully porous particles on a KNAUER preparative HPLC system equipped with a binary pump (50 mL head pump), two semi-preparative 3 mm UV flow cells, an 8-port-2-positions valve (recycling valve), a 6-port-2-positions valve (injection valve) and a fraction collector Foxy R1, as schematically shown in Fig. 1. Data acquisition, data handling and instrument control were performed by PurityChrom CDS software.

The semi-preparative recycling method was performed under isocratic conditions with 100% EtOH as mobile phase, permitting the direct injection of the *Cannabis* extract without any treatment.

The flow rate was 3.5 mL/min and the injection volume was 20 $\mu$L.

### Batch chromatography

Batch purification was performed on the same instrument used for the APR process, equipped with one column (150 $\times$ 8 mm C18). Three different procedures were performed: 1) the same experimental conditions as APR (flow rate = 3.5 mL/min, injection volume = 20 $\mu$L, mobile phase = 100% EtOH); 2) smaller injection volume to obtain quasi-baseline separation (flow rate = 3.5 mL/min, injection volume = 0.5 $\mu$L, mobile phase = 100% EtOH); 3) different mobile phase to obtain baseline separation (flow rate = 3.5 mL/min, injection volume = 20 $\mu$L, mobile phase = 80/20% EtOH/H_2_O).

Performance parameters (purity, recovery, productivity, solvent consumption) of the three batch methods were calculated and compared to APR process.

## Results and discussion

**Fig. 2:**
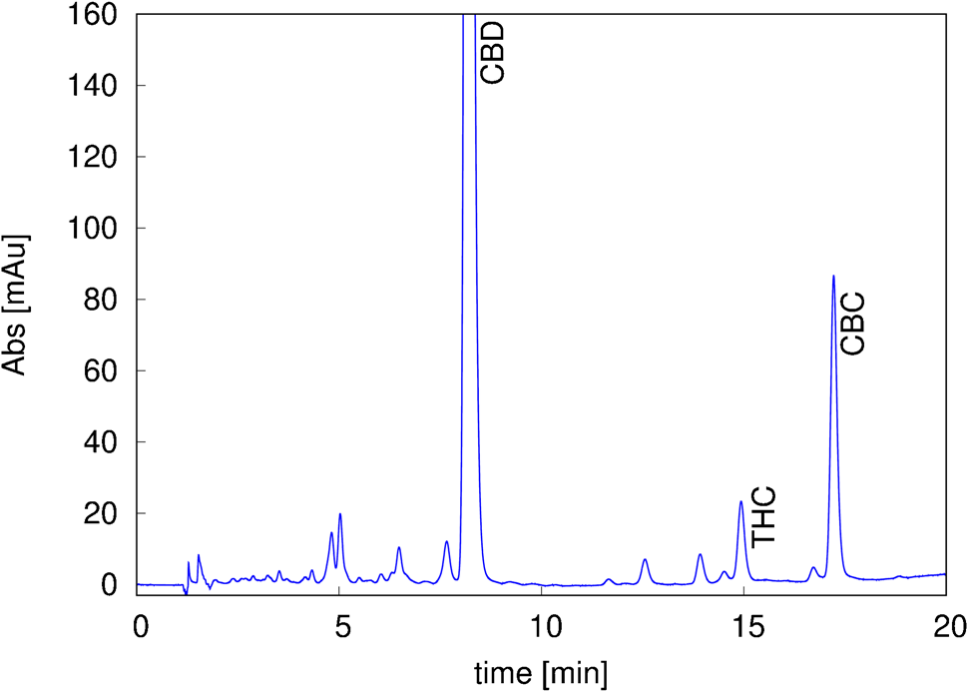
Analytical chromatogram of *Cannabis* extract obtained with gradient method (see “Offline analysis”). CBD: cannabidiol, THC: tetrahydrocannabinol, CBC: cannabichromene

The CBD-rich *Cannabis* sample was firstly characterised using the analytical method reported in “Offline analysis” to identify and quantify the main cannabinoids. Analytical chromatogram is reported in Fig. 2. As it can be clearly seen, CBD is the dominant species with a concentration of 11.3 mg/mL and 80.5% initial purity.

**Fig. 3:**
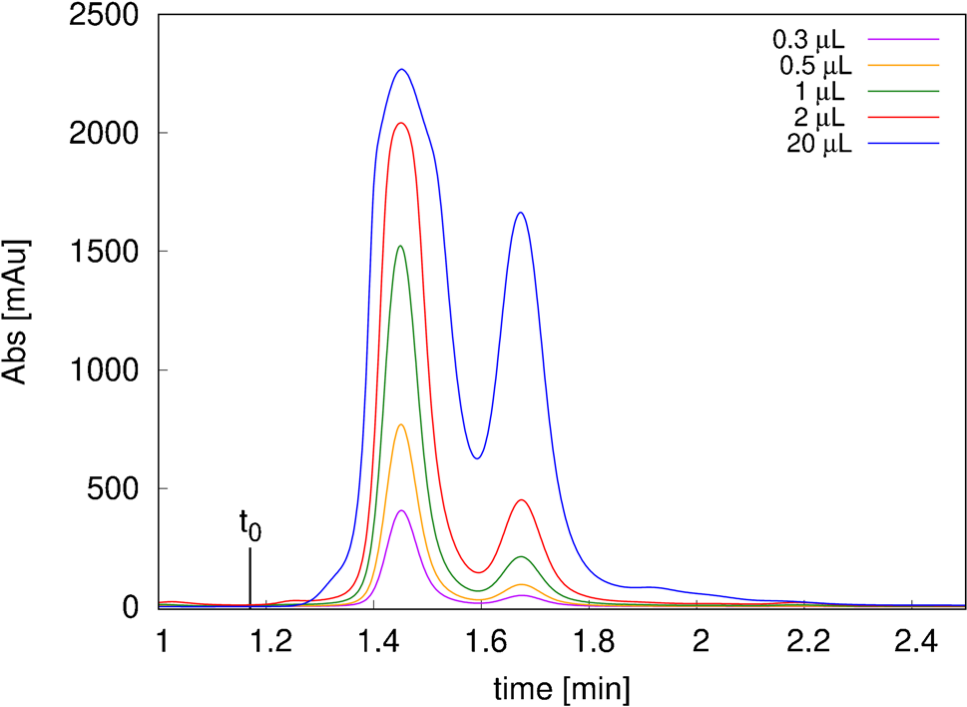
Overlay of chromatograms of *Cannabis* extract obtained with increasing injection volume (0.3, 0.5, 1, 2 and 20 $\mu$L) using 150$\times$8 mm column and isocratic elution at 100% EtOH. The first peak corresponds mainly to CBD and the second peak contains mainly CBC and THC

Figure 3 shows the starting batch chromatograms at increasing injection volumes obtained with 150$\times$8 mm C18 column using pure ethanol as mobile phase. As it can be noted, two main peaks, not fully resolved, are present. As already pointed out, compounds are only slightly retained due to the high elution strength of the mobile phase. Standard injections have indicated that the first eluting peak mainly contains CBD (i.e. the major component in the sample), while the second peak is made of a co-elution of CBC and THC (data not shown).

The peak shapes increased linearly with injection volumes, indicating a Gaussian behaviour of cannabinoids under study. At very small injection volumes the two groups of peaks are quasi-baseline resolved, while higher volumes lead to a more severe peak overlapping.

### Semi-preparative recycling chromatography

**Fig. 4:**
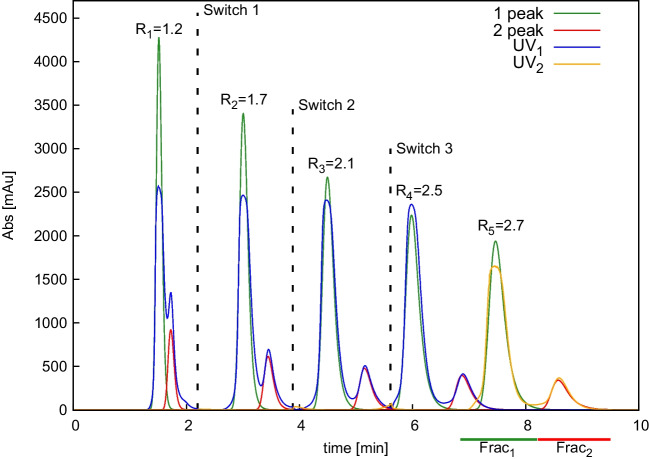
Overlay of simulated and experimental chromatograms from APR using C18 150$\times$8 mm columns. Green line: simulated first peak, red line: simulated second peak, blue line: experimental chromatogram from UV_1_, yellow line: experimental chromatogram from UV_2_. Theoretical resolution ($R_n$) was calculated through Eq. 1 for *n* = 1 and Eq. 2 otherwise

Prior to experimentally perform recycling chromatography, simulations have been carried out in order to predict the compound behaviour depending on the number of switches (or passages through *n* columns). To do so, CBD and THC standards and the *Cannabis* extract were injected on the ID 8 mm column. Peaks were fitted with EMG function (see Fig. S2) to obtain the parameters needed for the simulation ($t_R$, $\sigma$, $\tau$). Optimal agreement between parameter values was found by comparing standards and the real sample. Band profiles were then simulated after passage through *n* columns using a MATLAB code (Fig. S3). Results indicate that the maximum number of allowed column passages is *n* = 5, meaning $n_s$ = 3 switches, i.e. until overlapping between the second peak eluting from the upstream column and the first peak eluting from the downstream column has been observed (Fig. S3 at 7 min). Real sample was then injected into the recycling system, after having set in advance the maximum number of automatic switches to be performed ($n_s$= 3), based on simulation results. The peaks after 3 switches elute from column 2, with the valve in position B (see Fig. 1). The absence of new switches leads the flow to pass through column 1 and then to UV_2_. Hence, the sample experiences a total of $n_{tot}$ = 5 columns. An optimal agreement between simulated and experimental chromatograms was found, as reported in Fig. 4. Differences in peaks height are due to the saturation of the UV cell.

**Table 1:** Recovery (%) and Purity (%) from offline analysis of the two fractions collected with APR process

Fraction	CBD		$\Delta$ ^9^-THC	
	*Rec* (%)	*P* (%)	*Rec* (%)	*P* (%)
1	99.7%	97.3%	0.0%	0.0%
2	0.3%	3.1%	100.0%	32.2%

Finally, two fractions were collected through a fraction collector and analysed offline to calculate purity and recovery values of CBD and THC. Results are reported in Table 1. As it can be seen, fraction 1, related to the first eluted peak, contains highly pure CBD with almost 100% recovery, while fraction 2, related to the second eluted peak, contains the entire amount of THC. Analytical chromatograms of the two fractions are reported under SI (Fig. S4). This indicates that the recycling process was able to completely deplete THC from the CBD-rich fraction. It is worth pointing out that CBD purity does not reach 100% due to the presence of minor coeluting compounds present into the *Cannabis* extract. Nevertheless, obtaining 100% CBD purity was beyond the scopes of this work.

### Comparison of process performance between recycling and batch chromatography

In order to have a more complete overview of the performance of APR procedure, the outcomes of the separation were compared to those of traditional batch chromatography, since the latter is the most common technique used for *Cannabis* purification. Data are reported in Table 2.

**Table 2:** Performance parameters related to CBD of the different methods. A: APR process, B: Batch process at the same CBD purity as A, C: Batch process at the same CBD recovery as A, D: Batch process at the same CBD purity and recovery as A, E: Batch process at the same separation as A

Case	Method	$V_{inj}$	*MP*	Purity	Recovery	Productivity	Solvent consumption
		$\mu$L	% EtOH	%	%	mg_CBD_/h	mL/mg_CBD_
A	APR	20	100	97.3	99.7	1.6	135
B	Batch	20	100	97.1	69.1	3.9	54
C	Batch	20	100	84.5	99.9	5.6	45
D	Batch	0.5	100	97.4	99.6	0.1	1501
E	Batch	20	80	98.3	99.4	1.7	120

A first batch was performed keeping the same experimental conditions as the APR process (case A), i.e. injection volume of 20 $\mu$L and 100% EtOH as mobile phase. Fractions have been collected, as reported in Fig. S5, and analysed offline. Following the purity-yield trade-off, according to which recovery can only be increased by lowering purity and vice versa, the two extreme cases have been taken into account by pooling different fractions: the highest achievable purity scenario (case B) and the highest achievable recovery scenario (case C). On the one hand, in case B, the same purity obtained with APR (case A) was achieved but at the expense of recovery, which is 30% smaller. On other hand, case C refers to the full CBD recovery. In this situation, however, CBD fraction is contaminated with 13% pure THC and the final purity is 84%. Hence, case C does not fulfil the purpose of complete THC depletion.

Batch experimental conditions were then modified to provide the same purity and recovery values as the reference case A. To do so, in case D, following the chromatograms reported in Fig. 3, injection volume was decreased to 0.5 $\mu$L to provide adequate resolution between the two main peaks. In case E, 20% water was added in the mobile phase to increase retention and separation, keeping injection volume at 20 $\mu$L (Fig. S6).

All examined cases were also compared in terms of productivity and solvent consumption. The best results in terms of both productivity and solvent consumption are obtained with batch cases B and C. Nevertheless, in case B roughly 30% of the target product is lost, representing a possible limitation in case of highly valuable and expensive compounds, while, in case C the final product still contains some THC, hence the final scope is not satisfied. As expected, case D shows the worst performance due to the very low injected amount of sample. Comparable results to the reference case A are obtained with case E. It is worth noting that in method E the mobile phase is a mixture of EtOH and water. In this context, evaporation step for the preparation of the final commercial product may negatively affect the greenness of the process. Indeed, EtOH will be removed faster than water thanks to its lower boiling point, leading to a higher energy requirement with respect to case A.

Following data reported in Table 2, the bottleneck towards the complete greenness of APR process is the large amount of solvent used. This issue could be alleviated through the implementation of an additional valve to the system that permits the internal recycling of the solvent. In this way, the solvent eluting from the recycling circuit is re-injected into the system, until the last switch where the flow then is directed into the fraction collector. The adoption of this improved system could grant the same elution performance as the traditional APR process, but saving half of the solvent.

## Conclusions

In the last years, more and more attention is being paid to the environmental impact and greenness of chromatographic processes. The continuous research of sustainable techniques has led to the rebirth of methodologies developed decades ago. Among them, supercritical fluid chromatography, simulated moving bed and recycling chromatography are possibly the most interesting ones.

This study showcases the effectiveness of utilising a combination of green solvents, recycling chromatography, and theoretical modelling as an industrial strategy to purify *Cannabis* extracts, aiming to reduce the environmental footprint of traditional purification methods for high-value molecules.

### Supplementary Information

Below is the link to the electronic supplementary material.

Supplementary file 1 (pdf 2228 KB)
