# Supplementary material for: Sustainable cannabinoids purification through twin-column recycling chromatography and green solvents
Source: Anal Bioanal Chem. 2024 May 15;416(18):4091–9. doi: 10.1007/s00216-024-05332-7 (PMC11249647; doi:10.1007/s00216-024-05332-7)
Supplement: Supplementary file 1 — (pdf 2228 KB) [file 216_2024_5332_MOESM1_ESM.pdf]

SUPPLEMENTARY MATERIAL

Contents

1 MATLAB simulation code **3**

2 Offline analysis **6**

3 Batch chromatography **7**

3.1 100% EtOH 7

3.2 80% EtOH 7

1 MATLAB simulation code

A schematic representation of all the stages needed for the complete method development is reported in Fig. 1.

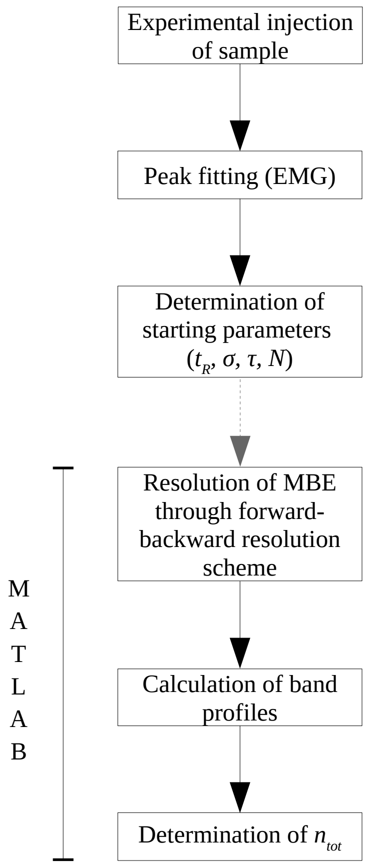

Fig. 1: Schematic representation of the method development steps.

More in detail, the parameters needed for the theoretical prediction of band profiles are: retention time (t_R), standard deviation (σ), time constant of exponential decay function (τ) and plate number (N). They have been obtained by fitting experimental peaks with an EMG function (see main text for details). As an example, the result of peak fitting applied to the *Cannabis* extract is reported in Fig. 2.

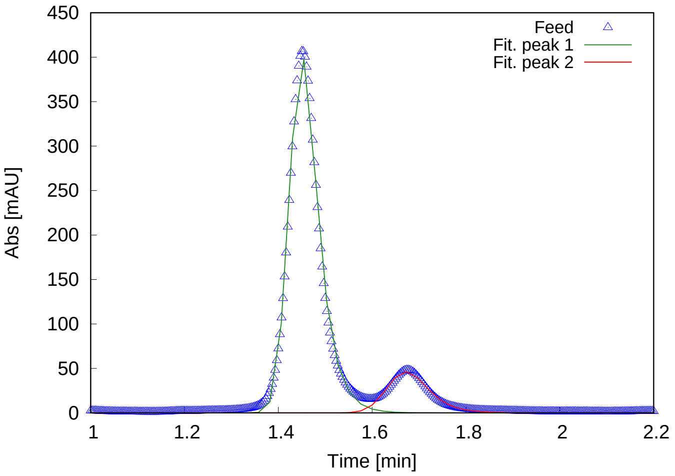

Fig. 2: Peak fitting with EMG function applied to *Cannabis* extract. First peak: CBD, second peak: CBC and THC.

Then a MATLAB code has been used for the calculation of band profiles in recycling chromatography, based on the number of column passages (n), using a forward-backward differences scheme for obtaining numerical solutions of the Equilibrium-Dispersive (ED) Model of chromatography [1] (see main text for details).
Results are reported in Fig. 3 for n=5 that corresponds to a number of switches n_s=3, since 2 columns are used. If the recycling process is performed on one column, only n=4 column passages are allowed in order to avoid the "re-mixing" of the two peaks. Otherwise, the use of APR (two columns) allows one more passage through column. Indeed, the two columns are connected in series, so what elutes from upstream column is directly injected into downstream column, without any switch, before leaving the system.

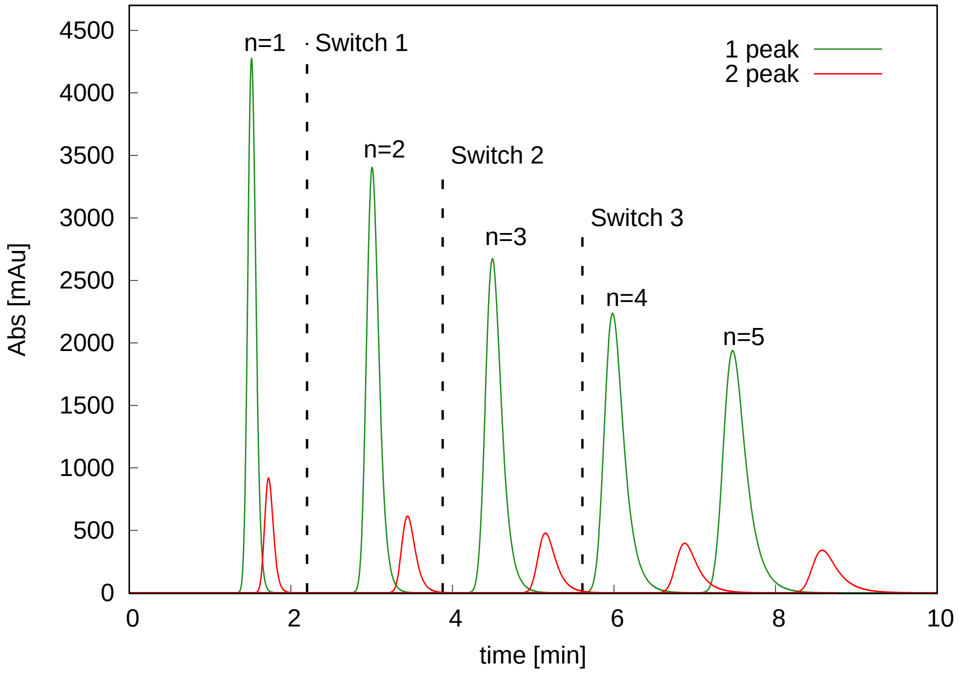

Fig. 3: Theoretical prediction of band profiles using MATLAB code for n=5.

2 Offline analysis

Fig. 4 shows offline analysis of recycling fractions using analytical method described in [5]. See Fig. 4 of the main text for the identification of the two fractions.

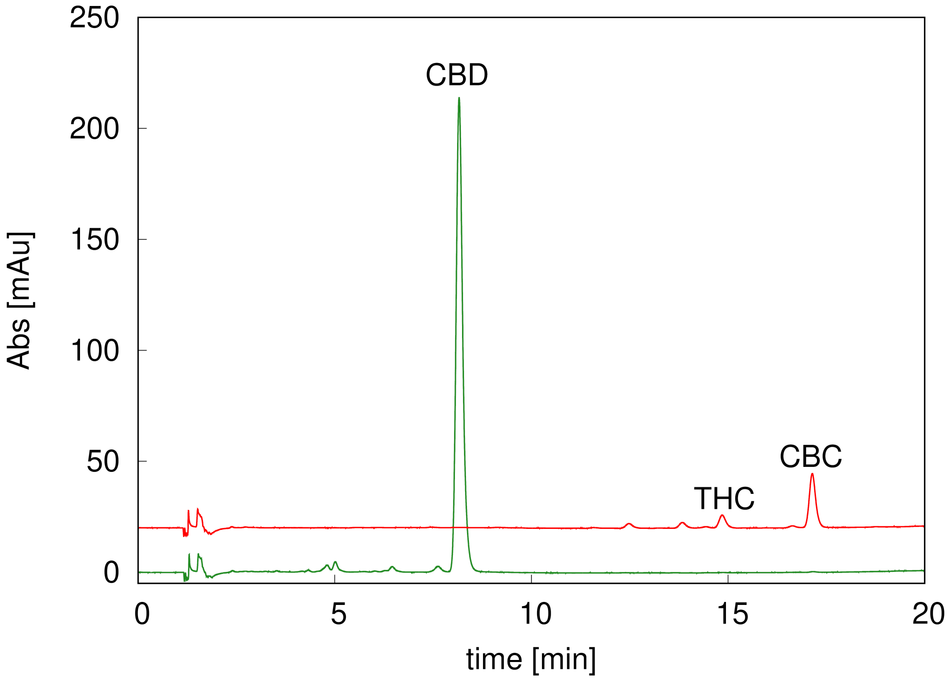

Fig. 4: Offline analysis of recycling fractions. In green the chromatogram of first fraction and in red the chromatogram of second fraction.

3 Batch chromatography

3.1 100% EtOH

Fig. 5 shows a batch chromatogram obtained by injecting 20 μL of *Cannabis* sample in a C18 150×8 mm column using 100% ethanol as mobile phase.
The fractions collected from the batch were analyzed offline using analytical method reported in [5]. Then, purity, recovery, productivity and solvent consumption have been calculated for cases B and C reported in Table 2 of the main text.

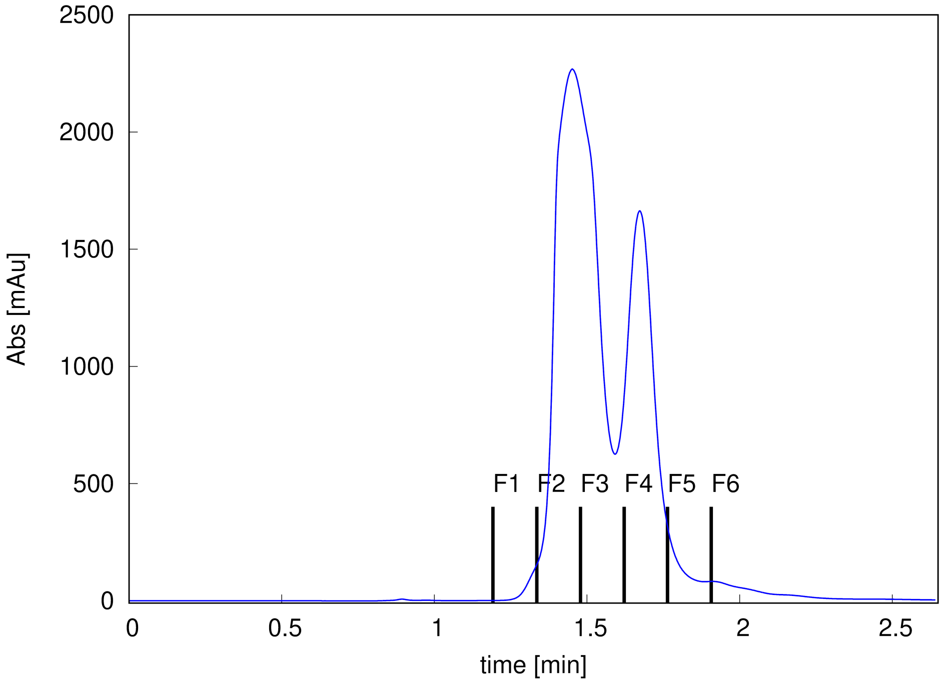

Fig. 5: Batch chormatogram of 20 μL injection of *Cannabis* sample using 100 % ethanol as mobile phase.. Collected fractions are indicated with increasing numbers (F1-F6).

3.2 80% EtOH

Fig. 6 shows a batch chromatogram obtained by injecting 20 μL of *Cannabis* sample in a C18 150×8 mm column using 80/20 % ethanol/water as mobile phase to obtain the baseline separation between the peaks of CBD and THC.
The CBD peak has been collected and purity, recovery, productivity and solvent consumption have been calculated for case E in Table 2 of the main text.

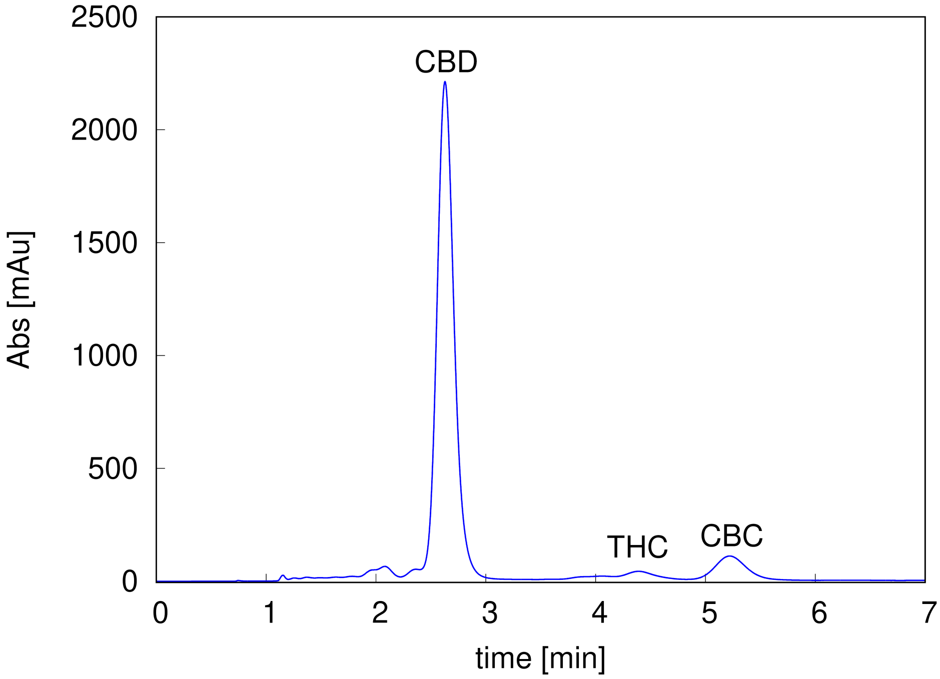

Fig. 6: Batch chormatogram of 20 μL injection of *Cannabis* sample using 80/20 % ethanol/water as mobile phase.

References

[1] G. Guiochon, A. Felinger, A. Katti, D. Shirazi, Fundamentals of Preparative and Nonlinear Chromatography, 2nd Edition, Academic Press, Boston, MA, 2006.

[2] R. T. Maruyama, P. Karnal, T. Sainio, A. Rajendran, Design of bypass-simulated moving bed chromatography for reduced purity requirements, Chem. Eng. Sci. 205 (2019) 401–413

[3] A. Rajendran, G. Paredes, M. Mazzotti, Simulated moving bed chromatography for the separation of enantiomers, J. Chromatogr. A 1216 (2009) 709–738

[4] Q-L. He, S. Leweke, E. von Lieres, Efficient numerical simulation of simulated moving bed chromatography with a single-column solver, Computers and Chemical Engineering 111 (2018) 183–198

[5] L. Loxterkamp, S. Stephan, K. Monks, (C)an(n)alyze: determination of 16 cannabinoids inside flowers, oils and seeds. (2019) Application note VPH0070, KNAUER Wissenschaftliche Geräte GmbH
